# Supplementary material for: A new class of bilayer kagome lattice compounds with Dirac nodal lines and pressure-induced superconductivity
Source: Nat Commun. 2022 May 19;13:2773. doi: 10.1038/s41467-022-30442-0 (PMC9120444; doi:10.1038/s41467-022-30442-0)
Supplement: Supplementary file 1 — Supplementary information [file 41467_2022_30442_MOESM1_ESM.pdf]

Supplementary information for "A new class of bilayer kagome lattice compounds with Dirac nodal lines and pressure-induced

Mengzhu Shi[1], Fanghang Yu[1], Ye Yang[1], Fanbao Meng[1], Bin Lei[1], Yang Luo[1], Zhe Sun[2], Junfeng He[1], Rui Wang[3], Zhicheng Jiang[4], Zhengtai Liu[4], Dawei Shen[4], Tao Wu[1,5], Zhenyu Wang[1,5], Ziji Xiang[1], Jianjun Ying[1,5] and Xianhui Chen[1,5,6,7]

[1]CAS Key Laboratory of Strongly-coupled Quantum Matter Physics, Department of Physics, University of Science and Technology of China, Hefei, Anhui 230026, China

[2] National Synchrotron Radiation Laboratory, University of Science and Technology of China, Hefei, Anhui 230029, China.

[3] Institute for Structure and Function & Department of physics & Center for Quantum Materials and Devices, Chongqing University, Chongqing 400044, China.

[4] State Key Laboratory of Functional Materials for Informatics, Shanghai Institute of Microsystem and Information Technology, Chinese Academy of Sciences, Shanghai 200050, China.

[5]CAS Center for Excellence in Superconducting Electronics (CENSE), Shanghai 200050, China.

[6]CAS Center for Excellence in Quantum Information and Quantum Physics, Hefei, Anhui 230026, China.

[7]Collaborative Innovation Center of Advanced Microstructures, Nanjing University, Nanjing 210093, China.

Supplementary Table 1. Crystal data and structure refinement for V_6Sb_4, RbV_6Sb_6 and CsV_6Sb_6 determined by single-crystal X-ray diffraction experiments.

Empirical formula	V_6Sb_4	RbV_6Sb_6	CsV_6Sb_6
Formula weight	792.64	1121.61	1169.05
Temperature/K	300		
Crystal system	trigonal		
Space group	$R\bar{3}m$ (No. 166)		
a/Å	5.5857(2)	5.50650(10)	5.5123(2)
b/Å	5.5857(2)	5.50650(10)	5.5123(2)
c/Å	20.4301(6)	34.6121(9)	35.2781(10)
α/°	90		
β/°	90		
γ/°	120		
Volume/Å^3	552.02(4)	908.89(4)	928.33(7)
Z	3		
ρ_{calc}g/cm^3	7.153	6.148	6.273
μ/mm^{-1}	174.450	145.497	160.288
F(000)	1026.0	1443	1497
Crystal size/mm^3	0.11 × 0.09 × 0.03	0.11 × 0.12 × 0.04	0.15 × 0.15 × 0.04
Radiation	Cu $K\alpha$ (λ = 1.54184)		
2Θ range for data collection/°	18.852 to 130.232	19.306 to 133.142	15.068 to 158.56
Index ranges	$-6 \le h \le 6$, $-6 \le k \le 6$, $-16 \le l \le 24$	$-6 \le h \le 6$, $-6 \le k \le 6$, $-38 \le l \le 40$	$-6 \le h \le 5$, $-6 \le k \le 6$, $-44 \le l \le 44$
Reflections collected	1262	1887	3222
Independent reflections	139, R_{int} = 0.0719, R_{sigma} = 0.0299	239, R_{int} = 0.0868, R_{sigma} = 0.0329	292, R_{int} = 0.0854, R_{sigma} = 0.0297

Data/restraints/parameters	139/0/14	239/0/18	292/0/18
Goodness-of-fit on F^2	1.234	1.099	1.197
Final R indexes [I>=2σ (I)]	R_1 = 0.0557, wR_2 = 0.1328	R_1 = 0.0358, wR_2 = 0.0924	R_1 = 0.0361, wR_2 = 0.0950
Final R indexes [all data]	R_1 = 0.0589, wR_2 = 0.1353	R_1 = 0.0363, wR_2 = 0.0931	R_1 = 0.0368, wR_2 = 0.0957
Largest diff. peak/hole / e $Å^{-3}$	2.48/-1.79	2.53/-2.15	3.61/-2.96

Supplementary Table 2. Fractional atomic coordinates and equivalent isotropic displacement parameters ($Å^2$) for RbV_6Sb_6, CsV_6Sb_6 and V_6Sb_4.

Chemical formula	Atom (site)	x	y	z	U(eq)
RbV_6Sb_6	Sb1(6c)	0.333333	0.666667	0.80126(2)	0.0156(4)
	Sb2(6c)	0.666667	0.333333	0.73253(2)	0.0189(4)
	Sb3(6c)	0	0	0.73376(2)	0.0191(5)
	Rb1(3a)	0.333333	0.666667	0.666667	0.0406(7)
	V1(18h)	0.3422(2)	0.17112(12)	0.79836(3)	0.0162(5)
CsV_6Sb_6	Sb1(6c)	0.666667	0.333333	0.19802(2)	0.0090(4)
	Sb2(6c)	0.333333	0.666667	0.26541(2)	0.0118(4)
	Sb3(6c)	0	0	0.26424(2)	0.0125(4)
	Cs1(3a)	0.666667	0.333333	0.333333	0.0274(5)
	V1(18h)	0.17129(11)	0.3426(2)	0.20104(3)	0.0093(4)
V_6Sb_4	Sb1(6c)	0.6667	0.3333	0.50093(7)	0.0258(11)
	Sb2(6c)	0	0	0.61009(6)	0.0249(11)
	V1(18h)	0.4974(3)	0.5026(3)	0.60930(9)	0.0262(12)

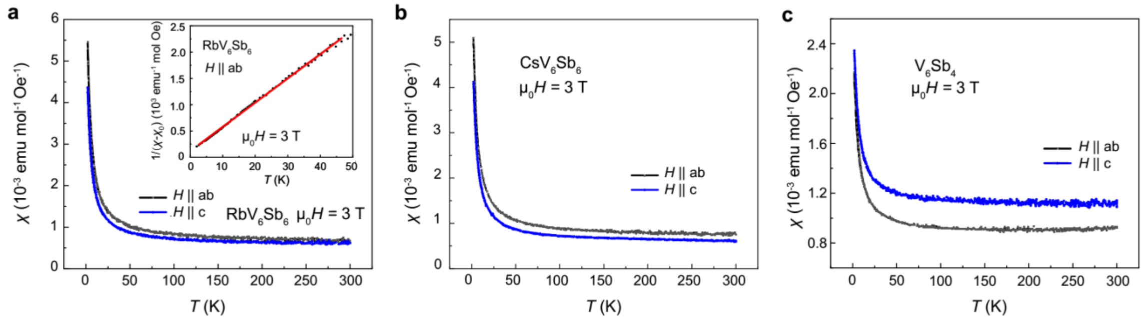

Supplementary Figure 1. The molar magnetic susceptibility χ of **a,** RbV_6Sb_6, **b,** CsV_6Sb_6 and **c,** V_6Sb_4 measured at $\mu_0 H = 3$ T plotted as a function of temperature. Data measured with magnetic field parallel to the *ab*-plane and *c*-axis are shown in black and blue, respectively. Inset of **a** shows the Curie-Weiss fit $\chi = \chi_0 + C/(T-\Theta)$ (red line) applied to the *ab*-plane susceptibility which is plotted as $1/(\chi - \chi_0)$ against the temperature T for $T < 50$ K. The fit yields a small Weiss temperature $\Theta = -2.5$ K and an effective magnetic moment 0.42 μ_B per chemical formula (corresponding to a 1.8% occupation of divalent V^{2+} ions on the V sites, which may be caused by defects in our crystals). The fitting results are consistent with a nonmagnetic state containing a small amount of defect/impurity-induced local moments.

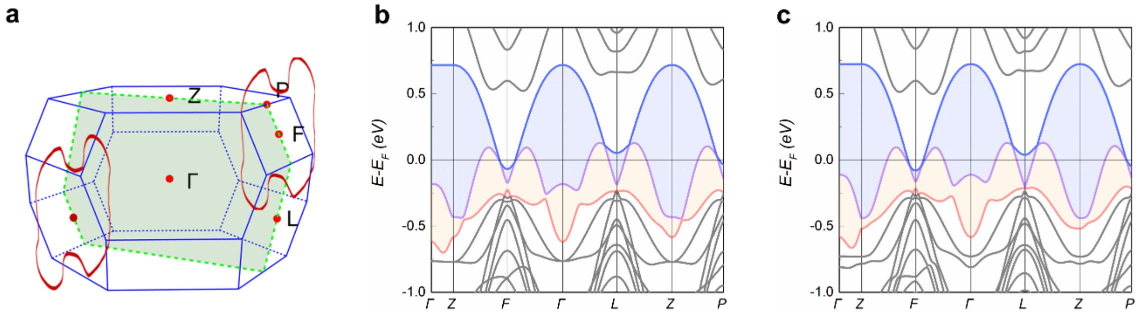

Supplementary Figure 2. **a,** The first Brillouin zone (BZ) of V_6Sb_4. The conduction band and valence band inverted at the F point (see the main text), forming the nodal rings (red colour) lying in the middle mirror plane of the BZ (green colour). The calculated band structures for **b,** RbV_6Sb_6 and **c,** KV_6Sb_6.

Supplementary Table 3. The parity at TRIMs and the Z_2 invariant for CsV_6Sb_6 and V_6Sb_4.

	Occ. band	Irrep.				Parity (prod.)				Invar.
		Γ (*D3d*)	F (C*2h*)	L (*D2h*)	Z (D3d)	δ_Γ	δ_F	δ_L	δ_Z	Z_2
CsV_6Sb_6	123	$\Gamma_5^+\oplus\Gamma_6^+$	$\Gamma_3^+\oplus\Gamma_4^+$	$\Gamma_3^-\oplus\Gamma_4^-$	$\Gamma_5^-\oplus\Gamma_6^-$	-	+	+	-	(0;111)
	121	Γ_4^+	$\Gamma_3^-\oplus\Gamma_4^-$	$\Gamma_3^+\oplus\Gamma_4^+$	Γ_4^-	-	+	-	+	(0;111)
	119	Γ_4^-	$\Gamma_3^+\oplus\Gamma_4^+$	$\Gamma_3^-\oplus\Gamma_4^-$	Γ_4^+	-	-	-	-	(0;000)
	117	Γ_4^+	$\Gamma_3^+\oplus\Gamma_4^+$	$\Gamma_3^-\oplus\Gamma_4^-$	Γ_4^-	+	-	+	-	(0;111)
	115	Γ_4^-	$\Gamma_3^-\oplus\Gamma_4^-$	$\Gamma_3^+\oplus\Gamma_4^+$	Γ_4^+	+	-	-	+	(0;111)
	113	$\Gamma_5^-\oplus\Gamma_6^-$	$\Gamma_3^-\oplus\Gamma_4^-$	$\Gamma_3^+\oplus\Gamma_4^+$	$\Gamma_5^+\oplus\Gamma_6^+$	-	+	-	+	(0;111)
	111	Γ_4^-	$\Gamma_3^+\oplus\Gamma_4^+$	$\Gamma_3^-\oplus\Gamma_4^-$	Γ_4^+	+	-	-	+	(0;111)
V_6Sb_4	103	Γ_4^-	$\Gamma_3^-\oplus\Gamma_4^-$	$\Gamma_3^-\oplus\Gamma_4^-$	$\Gamma_5^+\oplus\Gamma_6^+$	+	-	+	+	(1; 000)
	101	$\Gamma_5^-\oplus\Gamma_6^-$	$\Gamma_3^-\oplus\Gamma_4^-$	$\Gamma_3^+\oplus\Gamma_4^+$	Γ_4^+	-	+	-	+	(0; 111)
	99	Γ_4^-	$\Gamma_3^+\oplus\Gamma_4^+$	$\Gamma_3^+\oplus\Gamma_4^+$	Γ_4^-	+	-	-	+	(0; 111)
	97	Γ_4^-	$\Gamma_3^-\oplus\Gamma_4^-$	$\Gamma_3^+\oplus\Gamma_4^+$	$\Gamma_5^-\oplus\Gamma_6^-$	-	-	-	-	(0; 000)
	95	$\Gamma_5^-\oplus\Gamma_6^-$	$\Gamma_3^+\oplus\Gamma_4^+$	$\Gamma_3^-\oplus\Gamma_4^-$	Γ_4^-	+	+	-	+	(1; 111)
	93	Γ_4^-	$\Gamma_3^-\oplus\Gamma_4^-$	$\Gamma_3^+\oplus\Gamma_4^+$	Γ_4^+	-	+	+	-	(0; 111)
	91	Γ_4^+	$\Gamma_3^-\oplus\Gamma_4^-$	$\Gamma_3^-\oplus\Gamma_4^-$	$\Gamma_5^-\oplus\Gamma_6^-$	+	+	+	-	(0; 111)

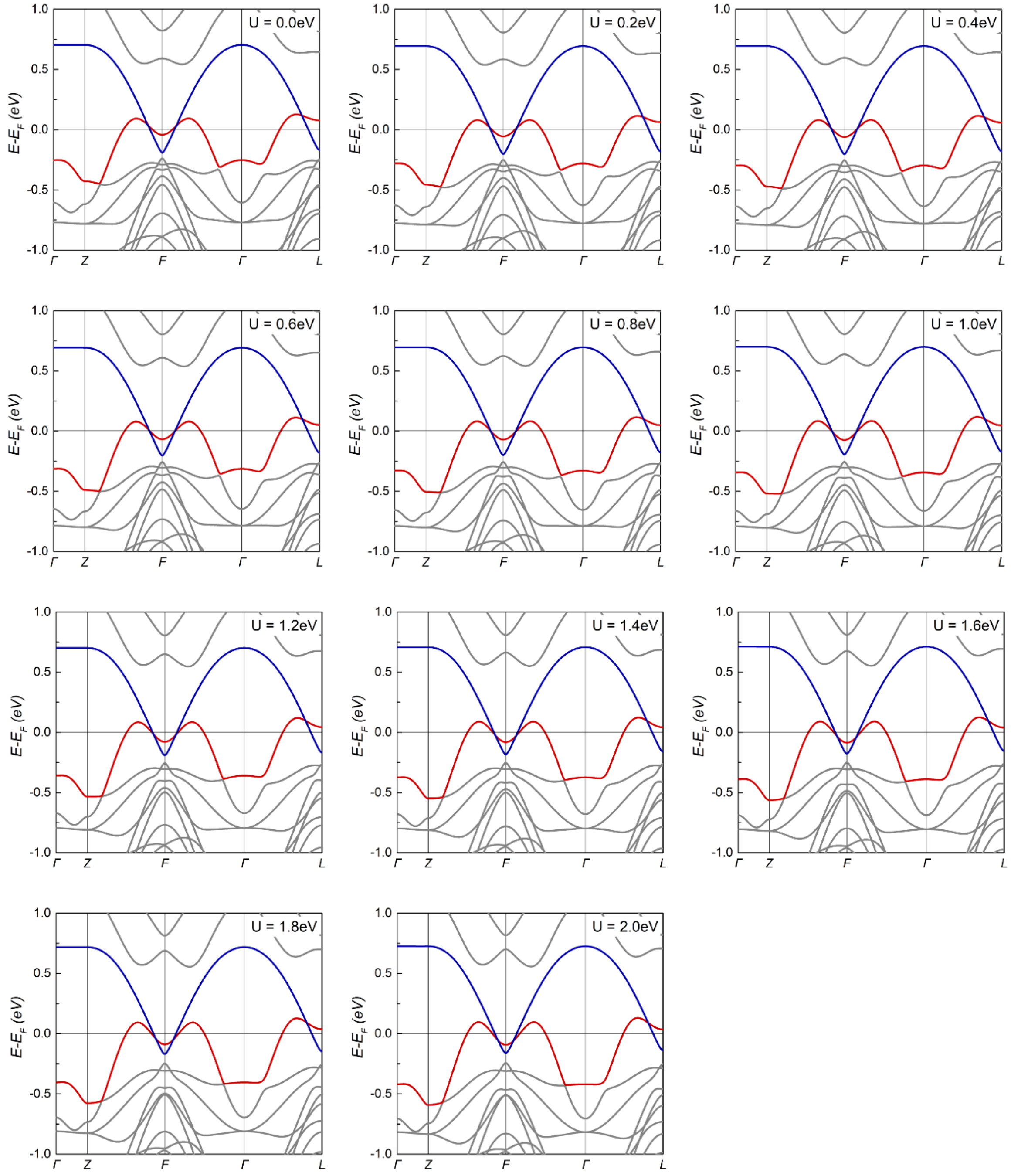

Supplementary Figure 3. The band structures of CsV_6Sb_6 vary with *U* values from 0 to 2 eV. The calculated results confirm that different *U* values only slightly affect the band profiles; band crossings and, in particular, the type-II nodal lines are robust against the on-site Coulomb correlation of V 3d orbitals. Here, two crossing bands near the Fermi level are colored by red and blue.

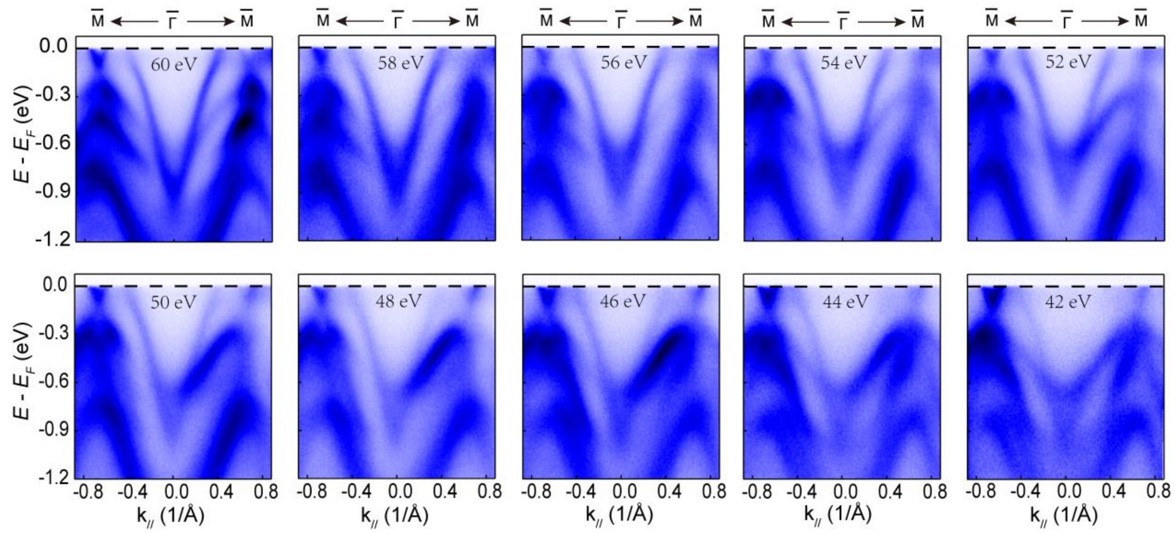

Supplementary Figure 4. ARPES intensity plots along the $\bar{\Gamma}-\bar{\mathrm{M}}$ momentum of CsV_6Sb_6 measured with varying photon energies from 42 to 60 eV at $T \approx 12$ K. A weak k_z-dependence of the bulk bands is found, which is consistent with the DFT calculations (Fig. 3g).

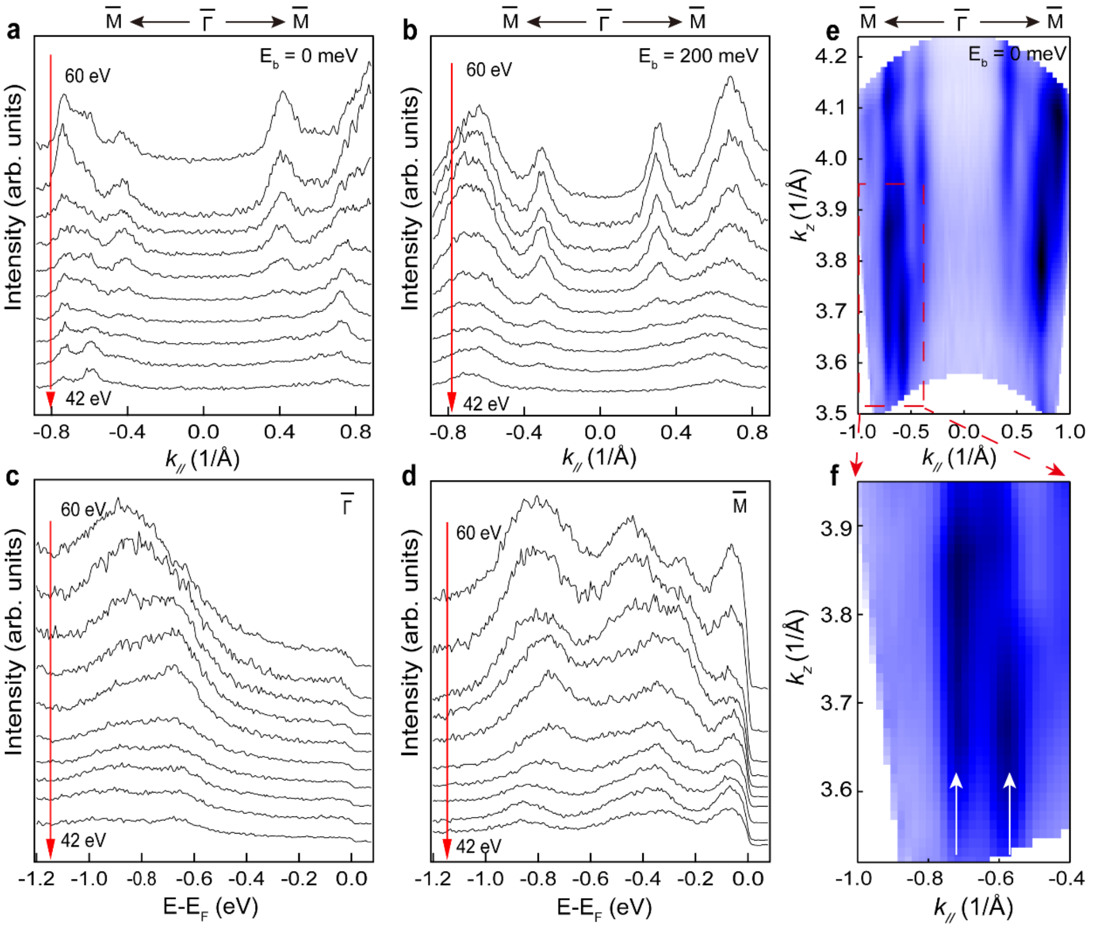

Supplementary Figure 5. The evolution of momentum distribution curves with varying photon energy from 60 eV to 42 eV at **a,** $E_b = 0$ (Fermi level) and **b,** $E_b = 200$ meV. The energy distribution curves measured with different photon energies at **c,** the $\bar{\Gamma}$ point and **d,** the $\bar{M}$ point. The curves in **a**-**d** are shifted vertically for clearance. **e,** Photon-energy dependent ARPES measurements of constant energy contour in k_z-k_x plane at the Fermi level. **f,** An expanded view showing the likely positions of the nodal lines formed by type-II Dirac cones along k_z (indicated by the white arrows).

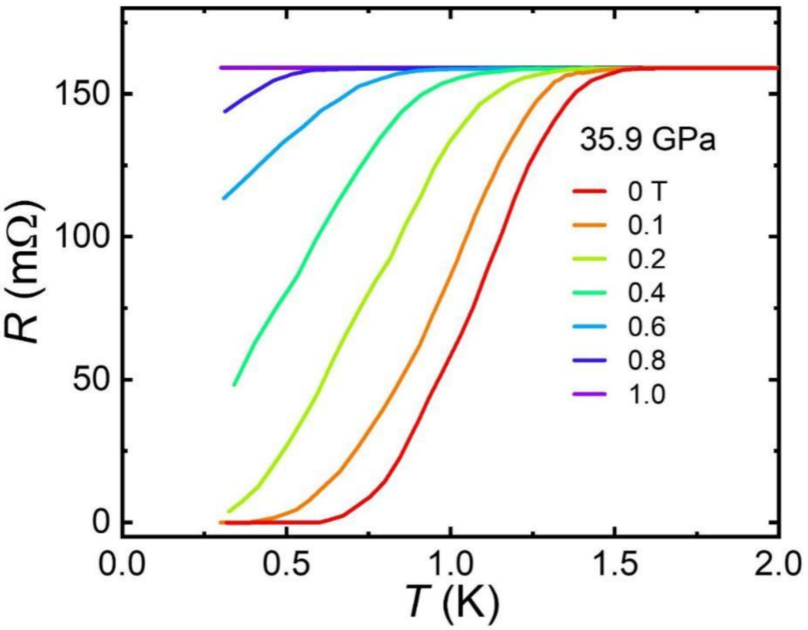

Supplementary Figure 6. Temperature dependence of the resistance of CsV_6Sb_6 measured at P = 35.9 GPa. The zero field curve shows a superconducting transition with an onset of ~1.4 K. By applying magnetic fields, the transition is gradually suppressed to lower temperatures. Under a magnetic field $\mu_0 H$ = 1 T, no superconductivity can be seen down to 300 mK. Such field sensitivity excludes the possibilities that the resistivity drop is due to a structural/magnetic transition instead of superconductivity.

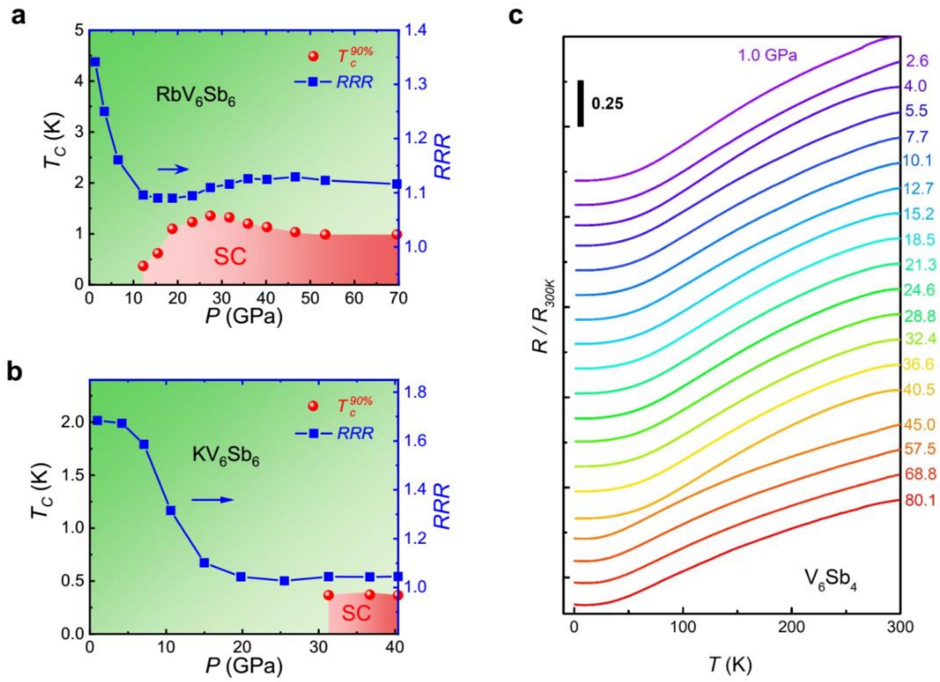

Supplementary Figure 7. Phase diagrams for **a,** RbV_6Sb_6 and **b,** KV_6Sb_6 under pressure. The superconducting transition temperature T_c (red solid circles) is determined as the temperature where the resistance drops to 90% of the normal state value. The maximum T_c is 1.36 and 0.37 K for RbV_6Sb_6 and KV_6Sb_6, respectively. **c,** Temperature dependence of the resistance of V_6Sb_4 normalized using the room temperature (300 K) value under various pressures up to 80.1 GPa. Data are shifted vertically for clearance. The black vertical bar denotes a scale of 0.25. No superconductivity can be detected down to 50 mK. The curves for $P > 41$ GPa were measured in a second sample.

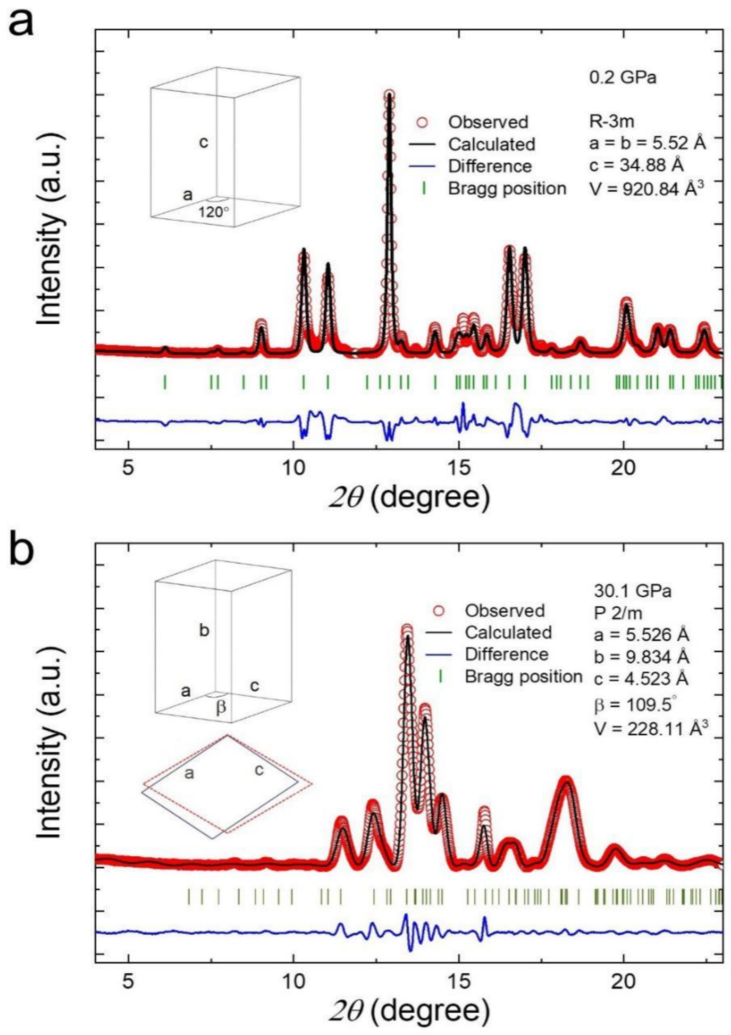

Supplementary Figure 8. The Le Bail fits for the XRD patterns measured under applied pressure of **a,** 0.2 GPa and **b,** 30.1 GPa. At 0.2 GPa, the data can be well fitted by the rhombohedral structure (space group R$\overline{3}$m) with $a = b = 5.52$ Å and c= 34.88 Å, consistent with the ambient pressure structure shown in Table S1. At 30.1 GPa, however, the XRD patterns is most sufficiently described by a monoclinic structure with the space group P2/m. This monoclinic structure can be viewed as a distorted original rhombohedral structure (see the sketches in the insets of each panel). The lattice parameter a in the rhombohedral structure develops to two unequal values a and c in the monoclinic phase. The angle β between the two in-plane basis vectors decreases from 120° in the rhombohedral phase to 109.5° in the monoclinic phase. A comparison of the in-plane rhombohedral unit cell (red dashed lines) and the monoclinic (black solid lines) unit cell is illustrated in the bottom inset of **b**.

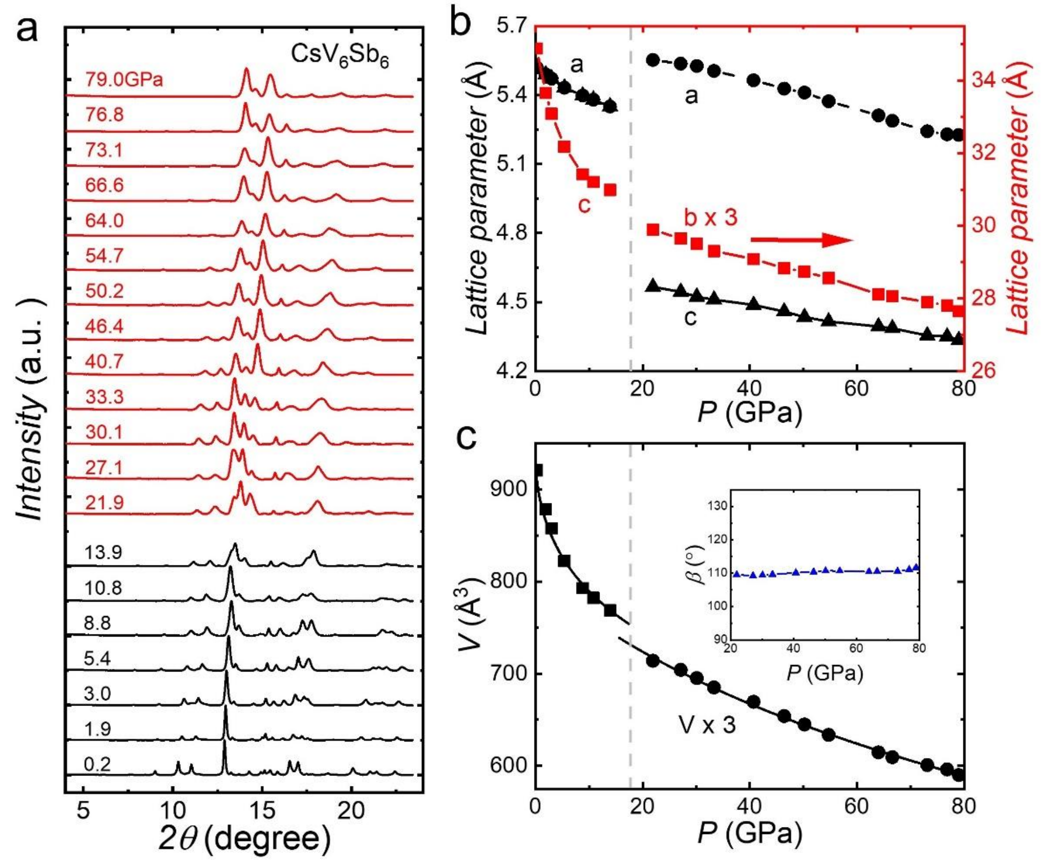

Supplementary Figure 9. **a**, XRD patterns measured in a CsV_6Sb_6 single crystal under high pressure up to 79 GPa with an incident wavelength λ = 0.6199 Å. A structural transition occurs between 13.9 and 21.9 GPa, above which the structure changes to monoclinic (XRD spectra highlighted by red color). **b,** The pressure dependence of the lattice parameters for CsV_6Sb_6. Above the structural transition (vertical gray dashed line), the two in-plane basis vectors (*a* and *c*, see the inset of Fig. S8b) become unequal. Note that the parameter *c* is defined on different basis vectors in the rhombohedral and monoclinic phases. **c,** The derived unit cell volume as a function of pressure for CsV_6Sb_6. The inset shows the negligible pressure dependence of the angle β (see the inset of Fig. S8b) in the monoclinic phase.
